# 4329 Investigating the role of Klotho in neurocognitive outcomes, brain volumes, and white matter changes in pediatric brain tumor survivors

**DOI:** 10.1017/cts.2020.305

**Published:** 2020-07-29

**Authors:** Caleb Simpeh Edwards, Schuyler Stoller, Sol Savchuk, Christian Rodrigo Ugaz Valencia, Liz Tong, Andreas Rauschecker, Dena Dubal, Cassie Kline, Sabine Mueller

**Affiliations:** 1 University Of California, San Francisco

## Abstract

OBJECTIVES/GOALS: Klotho is a protein linked to improved cognition in aging adults. A specific *KLOTHO* gene variant, KL-VS, increases circulating levels of Klotho. The current study aims to identify if the KL-VS haplotype and Klotho levels are associated with improved neurocognition in pediatric brain tumor survivors. METHODS/STUDY POPULATION: We are actively accruing pediatric brain tumor patients at UCSF alongside an existing multi-institutional cohort study investigating radiation-induced vasculopathies and cognitive outcomes in this population. Normal controls are being enrolled in parallel. Each patient undergoes: 1) single nucleotide polymorphism genotyping to identify KL-VS haplotype status, 2) enzyme-linked immunosorbent assays to measure circulating Klotho, 3) neurocognitive assessments with a computer-based, validated Cogstate battery, and 4) brain volume and white matter lesion segmentation analyses using MRI sequences obtained as part of routine care. RESULTS/ANTICIPATED RESULTS: Genotyping has been performed on 99 enrolled patients. KL-VS heterozygosity was seen in 22.7% of patients. To date, KL-VS status is not associated with neurocognitive outcomes at baseline or Year 1 testing. Association between KL-VS status, circulating Klotho levels, neurocognitive outcomes, brain volume and white matter lesion segmentation analyses is ongoing. We hypothesize that elevated Klotho levels will be associated with improved neurocognition, increased brain volumes in regions of interest and decreased white matter lesion volumes. DISCUSSION/SIGNIFICANCE OF IMPACT: If circulating Klotho leads to improved neurocognition in pediatric brain tumor survivors, Klotho levels might serve as a prognostic biomarker. Furthermore, as Klotho is being investigated for therapeutic indications, it may represent an intervention to prevent cognitive deficits in these patients.

